# Development of Liquid-Phase Plasmonic Sensor Platforms for Prospective Biomedical Applications

**DOI:** 10.3390/s24010186

**Published:** 2023-12-28

**Authors:** Sezin Sayin, You Zhou, Sheng Wang, Andres Acosta Rodriguez, Mona Zaghloul

**Affiliations:** 1 Department of Electrical and Computer Engineering, School of Engineering and Applied Science, The George Washington University, Washington, DC 20052, USA; 2 Department of Biomedical Engineering, School of Engineering and Applied Science, The George Washington University, Washington, DC 20052, USA; 3 3D Enviro, Barboursville, VA 22923, USA; andresa@3denviro.us

**Keywords:** localized surface plasmon resonance, biosensor, plasmonic sensor, biomedical diagnostics, nanosensors, liquid-phase diagnostics

## Abstract

Localized Surface Plasmon Resonance (LSPR) is an optical method for detecting changes in refractive index by the interaction between incident light and delocalized electrons within specific metal thin films’ localized “hot spots”. LSPR-based sensors possess advantages, including their compact size, enhanced sensitivity, cost-effectiveness, and suitability for point-of-care applications. This research focuses on the development of LSPR-based nanohole arrays (NHAs) as a platform for monitoring probe/target binding events in real time without labeling, for low-level biomolecular target detection in biomedical diagnostics. To achieve this objective, this study involves creating a liquid-phase setup for capturing target molecules. Finite-difference time-domain simulations revealed that a 75 nm thickness of gold (Au) is ideal for NHA structures, which were visually examined using scanning electron microscopy. To illustrate the functionality of the liquid-phase sensor, a PDMS microfluidic channel was fabricated using a 3D-printed mold with a glass slide base and a top glass cover slip, enabling reflectance-mode measurements from each of four device sectors. This study shows the design, fabrication, and assessment of NHA-based LSPR sensor platforms within a PDMS microfluidic channel, confirming the sensor’s functionality and reproducibility in a liquid-phase environment.

## 1. Introduction

A surface plasmon (SP) is the collective oscillation of conduction band electrons arising from the interaction of light with the conduction electrons of a metal. Photons and electrons couple to form a hybrid between a light wave and an excited electronic level. Hence, an enhanced optical near-field at metallic surfaces or within metallic nanostructures, a localized surface plasmon (LSP), occurs. The energy of surface plasmons is dependent on the excitation, the nucleus–electron interaction, features, material composition, and number of structures [1]. In a typical surface plasmon resonance (SPR) sensor, a transducing medium interrelating the optical and chemical parts and an electronic system for the optoelectronic part and data processing are present [2]. For instance, Houngkamhang et al. used an SPR imager and a multichannel flow cell based on the Kretschmann configuration and prism coupling for generating the SPR phenomena for blood-typing using an antibody array technique [3].

From the fundamental principles of surface plasmons to the distinctive advantages of localized surface plasmons, it is evident that LSPR sensors offer a novel and powerful approach to biosensing. LSPR, compared to SPR, can generate highly concentrated and intensified electric fields near the interface, thereby achieving remarkable confinement. What sets LSPR apart is its independence from coupling elements like prisms or angular detection, primarily due to the substantial scattering and absorption coefficients of LSPR-supporting nanostructures. This obviates the necessity for optical coupling elements or constraints related to polarization. LSPR exhibits spectral shifts that can span tens of nanometers. In comparison to SPR, LSPR boasts greater surface sensitivity attributed to a shorter field decay length of 10–30 nm while being less susceptible to variations in bulk refractive index and fluctuations in temperature. Table S1 illustrates a selection of plasmonic sensors, highlighting the materials utilized, sensor performance, and the applications. Consequently, LSPR-based biosensing systems can discern analytes near their surfaces without interference from distant molecules within the analyte solution. They can be excited by unpolarized light and do not require complex optics. The notably short evanescent field penetration into the surrounding dielectric medium holds the promise of enhanced miniaturization and multiplexed detection [4].

LSPR-based sensors offer real-time, label-free monitoring of two binding partners interacting. They can also be used to gather information on specificity, association and dissociation kinetics, and binding affinity. Nowadays, this technique is utilized in drug discovery, antibody characterization, proteomics, immunogenicity, food analysis, and biosensors in general, as they can detect very low concentrations of chemical and biological species near the surface [5].

Akib et al. fabricated a highly sensitive graphene-based multilayer (BK7/Au/PtSe2/Graphene) SPR biosensor using total internal reflection (TIR) to detect ligand-analyte immobilization in real time. In this system, Goos-Hänchen (GH) shift detection was utilized to improve the sensitivity with 2D nanomaterials [6]. In another study, Uddin et al. presented silicon and BaTiO3 on top of a Ag layer based on the Kretschmann configuration for biomedical diagnostics. This structure revealed a 7.6-fold improvement in sensitivity compared to the basic Kretschmann setup [7]. Qiu et al. fabricated a combined plasmonic photothermal (PPT) effect and LSPR-based biosensor for COVID-19 detection. They used gold nanoislands (AuNIs) functionalized with DNA receptors. Localized PPT increases in situ hybridization temperatures and helps distinguish two gene sequences that are almost identical [8]. Yang et al. fabricated a human angiotensin-converting enzyme 2 protein (ACE2)-functionalized silver nanotriangle (AgNT) array LSPR sensor for rapid detection. The detection limit was achieved in less than 20 min [9]. Yang and Li demonstrated nanohole arrays with a 630 nm period and a 320 nm hole diameter and investigated the sensitivity at different incident light angles [10]. Zhou et al. conducted an extensive study on nanohole array-based plasmonic sensors by fabricating arrays of different sizes and combining them with a PMMA-based microfluidic channel for use in biomarker detection [11].

Building on the versatile capabilities of LSPR-based sensors in various fields, their integration with microfluidic technology has shown promising potential for biomedical applications. Microfluidics enables the miniaturization of sensor systems in biology and chemistry, allowing the control and measurement of sample volumes. The capabilities of LSPR sensors are enhanced by integrating the sensing system with the microfluidic channel [12]. Incorporating SPR sensing technology into microfluidics offers several benefits, including automation, minimal sample usage, swift processing, and increased sensing effectiveness when appropriately designed. Microfluidics has the potential to accelerate reaction rates, shorten diffusion times, and improve surface regeneration efficiency. The automation enabled by microfluidics enhances reproducibility and provides precise control over every stage of the reaction process [13]. Zhou et al. produced nanocrescent arrays for use as a plasmonic sensor. These arrays were subsequently combined with a microfluidic system that incorporated two inlet channels; one channel supplied a sodium chloride solution, while the other introduced deionized water at varying flow rates. This setup allowed the formation of sodium chloride solutions with different surface concentrations in the detection region [14]. Chen et al. developed an integrated LSPR sensor and automated microfluidic control system for detecting inflammatory biomarkers in a label-free, multiplex manner. This system allows multiple reagents to be automatically directed, loaded, and switched through a customized program. As a demonstration, they successfully detected IgG and CRP in a multiplex fashion at various concentrations using this device [15].

This study embodies innovation in biosensor technology by focusing on the development, fabrication, and assessment of NHA-based LSPR sensor platforms integrated into a PDMS microfluidic channel. NHA sectors were simulated, designed, and fabricated. A novel liquid-phase setup was introduced for capturing target molecules, and a custom PDMS microfluidic channel was fabricated using 3D-printing technology to facilitate precise sample handling and reflectance-mode measurements across four device sectors. The successful integration of NHA-based LSPR sensors into a liquid-phase environment signifies their functionality and consistent reproducibility, marking a significant advancement in biosensing technology with promising implications for biomedical diagnostics.

## 2. Materials and Methods

### 2.1. NHA Design and Fabrication

To achieve suitable plasmonic structures for the sensor platforms, the specifications for the nanohole array (NHA) were carefully devised. Previous research has already investigated the design parameters for NHAs. Based on computational simulations, it was determined that NHAs with a diameter of 200 nm and a period of 400 nm provided the most favorable results. Each sensor platform comprised four NHA sectors measuring 300 μm × 300 μm, with each sector separated by 1 mm [16].

The deposition thickness of Au in a nanohole array significantly impacts the sensor performance. Changes in Au thickness modify plasmonic properties, including resonance wavelength and sensitivity, which affect the sensor’s detection abilities. Modifying the thickness of Au can enhance sensitivity by allowing greater penetration of the evanescent field through nanohole arrays, but it can also disrupt the plasmonic response [17]. Therefore, optimizing the Au deposition thickness is an important step before fabrication. To simulate the deposition of Au thickness on NHA structures, we utilized the Lumerical finite-difference time-domain (FDTD) simulator; this simulation models components where the complex interaction of optical, electronic, and thermal phenomena is critical to performance. For the NHA simulations, a 100 nm Si_3_N_4_ layer was designated followed by a 5 nm Ti layer. On top of these layers, a Au layer was added and a hole was created in the shape of a circle. To investigate Au thickness, the simulation specifications involved using the sweep mode, varying the Au thickness from 25 nm to 125 nm. The optical properties for Si_3_N_4_ were obtained from Kischkat’s data, those for Ti from Palik’s, and those for Au from Johnson and Christy’s data. Regarding object meshes, a 5 nm mesh with a 5 nm buffer was selected. Mesh refinement was set as 0 because the coating material is metal. A plane wave light source along the *z*-axis ranging from 400 nm to 900 nm in wavelength was utilized. The simulation was conducted in FDTD 3 dimensions with a 1000 fs simulation time and 300 K simulation temperature. Twenty-point data were acquired in sweep mode.

**Figure 1:**
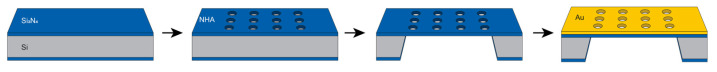
A schematic depicting the fabrication process for the plasmonic sensor platform, illustrating the patterning of NHA through EBL, the creation of a membrane window using a mask aligner and RIE, and the deposition of Au via an e-beam evaporator.

NHA devices were fabricated by depositing a 100 nm Si_3_N_4_ onto a 500 µm silicon substrate using low-pressure chemical vapor deposition (LPCVD), electron-beam lithography (EBL) patterning, and reactive ion etching (RIE). A membrane window on the backside was patterned using a mask aligner and RIE [18], as depicted in Figure 1. Five-nanometer Ti and seventy-five-nanometer Au films were deposited onto the surface using an electron-beam evaporator. The NHA structures were visualized using an FEI Teneo LV Field Emission Scanning Electron Microscope (SEM) at the George Washington University Nanofabrication and Imaging Center.

In contrast to the study by Yang and Li [10], our system features a smaller period and diameter in the nanohole arrays, resulting in a higher surface area-to-volume ratio. While their simulations focused on sensitivity investigations at various incident angles, the FDTD simulations conducted in this paper involved a vertical incident angle. Additionally, further simulations were executed to optimize the Au deposition thickness, ranging from 25 nm to 125 nm.

### 2.2. PDMS Well Fabrication

In aiming to apply plasmonic sensor platforms in liquid-phase biomedical diagnostic applications, the samples were intended to be introduced in a liquid state. To achieve this, a 3D fabricated mold was designed to create a containment vessel in the form of a PDMS channel. Among various options for fabricating microfluidic channels, such as PMMA and adhesives [11], PDMS was selected for its ease of fabrication, biocompatibility, flexibility, elasticity, ease of sealing, and optical transparency.

**Figure 2:**
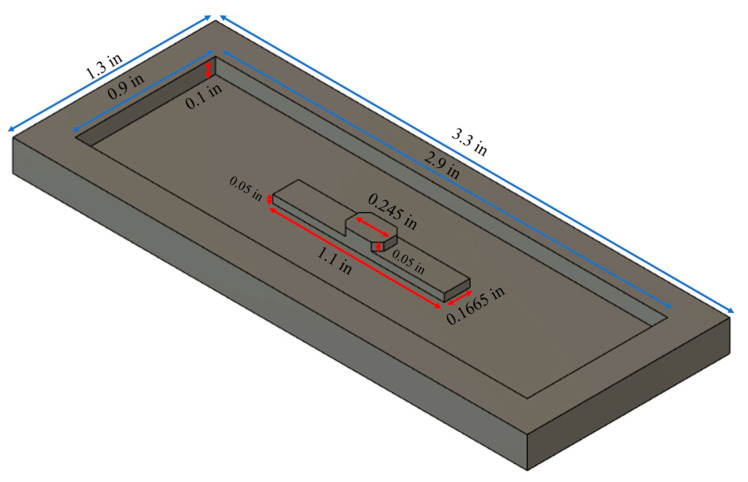
Dimensions of the 3D mold and sensor platform for PDMS fabrication.

The mold used for PDMS fabrication was created using the polymer solution casting technique. The PDMS casting mold was constructed from a ¼-inch thick PMMA stock (McMaster-Carr #8560K354, Elmhurst, IL, USA) using a CNC milling machine (MDA Precision, V8-TC8, Morgan Hill, CA, USA). The dimensions of the PDMS mold are depicted in Figure 2. Specifically, the dimensions of the sensor platform’s working area are 0.245 in length, 0.1665 in width, and 0.05 in height. The distance between the inlet and outlet openings is 1.1 in, while the thickness of the inlet and outlet channels is 0.05 in. 

**Figure 3:**
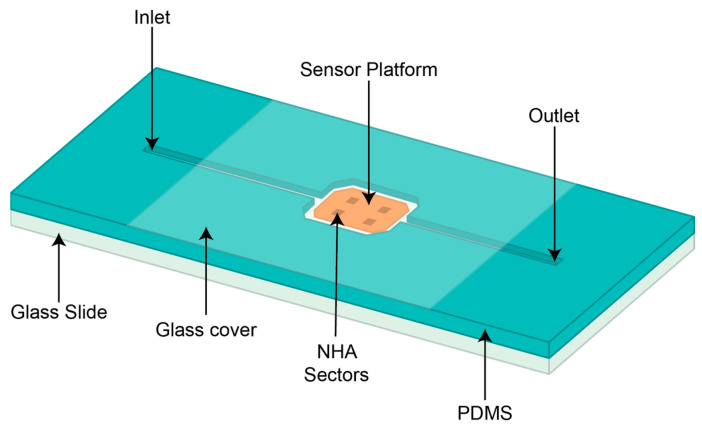
Illustration of the PDMS channel, including the liquid inlet and outlet, the sensor platform well, the underlying glass slide, and the upper glass cover.

This liquid-phase sensing platform consists of a PDMS base affixed to a glass slide, incorporating inlet and outlet channels for liquid introduction, as well as a sample chamber, as illustrated in Figure 3. This setup is specifically designed for integration with the spectrometer.

The PDMS channel fabrication process, involving mixing the PDMS base and curing agents at a ratio of 10:1, was performed at NIST CNST facilities. The resulting mixture was vigorously blended in a mixer at 2500 rpm for 3 min. To eliminate any trapped air bubbles, the mixture was poured into a prefabricated 3D-printed mold and subsequently placed within a desiccator for 15 min. Curing was achieved by subjecting the material to an oven at 80 °C for 2 h until solidification. Once solidified, it was securely attached to the 0.049-inch glass slide below and a 0.008-inch glass cover on top using an oxygen plasma bonding technique. The distance between the sensor’s working surface and the glass cover is 0.1 in, with 200 µL of liquid filling the area between the inlet and outlet passing through the sensor platform.

### 2.3. Spectrometer Setup and Measurements

The plasmonic sensing platform incorporates NHA structures designed to interact with visible light, inducing LSPR phenomena due to their subwavelength dimensions (200 nm) compared to the incident light’s wavelength. Vala et al. demonstrated the fabrication of NHAs placed within a refractive-index-symmetric setting, facilitating hybrid plasmonic modes and notable sensitivity to alterations in the refractive index near the surface, particularly within the nanoholes. In their setup, they exposed the sensing chip to a focused white light source, and the resultant reflected beam containing the SPR signal was directed through a beam splitter toward an imaging spectrometer [19]. Similarly, in this study, the design of the spectrometer setup allows the conduction electrons within the gold layer of the NHAs to polarize and aggregate on the NHA surface, leading to resonant oscillations. Alterations in the environment near these NHA features cause detectable changes in the LSPR characteristics. During response studies, variations in the estimated refractive indices with varied volume fractions of water–ethanol solutions result in shifts in the resonant wavelength and intensity changes in the LSPR that can be monitored by a spectrometer. The liquid sample made contact with the upper surface of the NHA within the PDMS channel.

**Figure 4:**
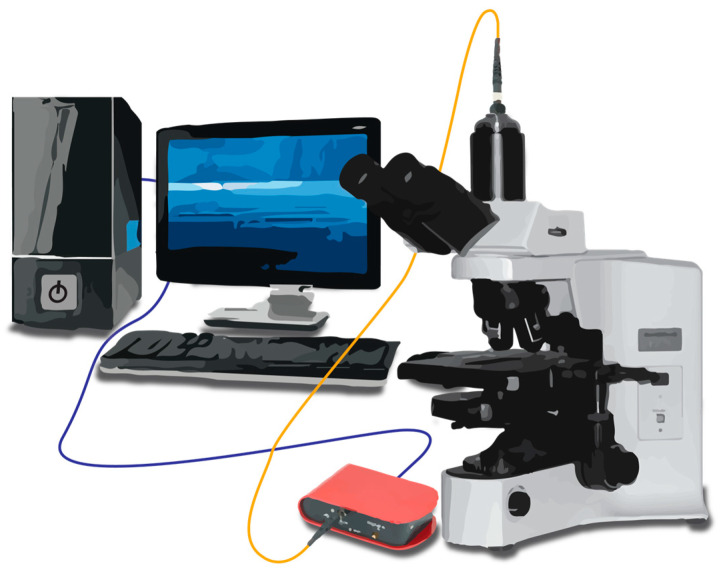
Visual representation of the optical setup for plasmonic sensor measurements, featuring a broad-spectrum visible light source, a microscope, a spectrometer, and an optical fiber.

To acquire optical spectra from the plasmonic sensor platforms, a reflectance-based bright-field spectrometer system was utilized. This system employed a broad-band visible light source (Motic MLC-150C, USA) to illuminate the sensor surfaces through a microscope (Motic PSM-1000, USA). The reflected light was captured by the spectrometer (Thorlabs CCS 200, USA) and a probe station (Signatone S-1160, USA) via an optical fiber. Figure 4 illustrates the optical setup for plasmonic sensor measurements, featuring a broad-spectrum visible light source, a microscope, a spectrometer, and an optical fiber capable of efficiently transmitting visible to near-infrared wavelengths connecting the spectrometer to the microscope. Before conducting sensing measurements, spectrometer calibration was performed to eliminate background signals originating from the Au surface and the broad-spectrum light source. Each sensor platform comprises 4 NHA sectors, as depicted in Figure 3. The 1 mm distance between each NHA array allows for conducting spectrometer measurements by focusing on each sector individually in different measurements. Spectral changes were measured by averaging 1000 spectra, followed by normalization with respect to the light source and dark noise. Parameters for the spectrometer setup, including the warm-up time of the light source, lamp power, sweep range, mode, and magnification, were optimized. The obtained results from the spectrometer measurements underwent processing and analysis using OriginPro software. This software was used for tasks such as data fitting, background subtraction, normalization, and determination of the position of the resonance peak from the raw spectrometer data. 

To evaluate the operational capabilities of the sensor within the newly designed sensor cell, a comprehensive series of tests was conducted. These tests encompassed various scenarios, ranging from simple air-to-water assessments to the examination of water–ethanol solutions with refractive indices estimated based on the research conducted by Arif et al. [20]. For each scenario, measurements were obtained from all four NHA sectors, and this procedure was repeated three times for each sector.

## 3. Results and Discussion

### 3.1. Au Evaporation Thickness Optimization and NHA Fabrication

To investigate the enhancement of the electric field, the primary focus was on transmission data as they provide insights into resonance peaks and their corresponding wavelengths. Based on the transmission results, it was determined that the (1 0) mode resonance peak associated with the nanoholes occurred at 599 nm. This resonance peak was notably prominent within the Au thickness range of 70–75 nm. In contrast, the (1 1) mode peak, relatively weaker, was observed at a wavelength of 450 nm. This specific peak tended to become more pronounced and wider as the thickness of the Au layer decreased, a trend generally considered unfavorable.

**Figure 5:**
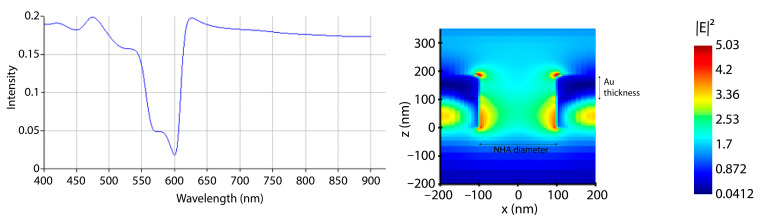
The outcomes of FDTD simulations for NHA structures with a 75 nm Au deposition. The transmission data (**left**) reveals a resonance peak at a wavelength of 599 nm, and the electric field enhancement within the NHA structure (**right**) indicates that the optimal Au thickness for e-beam evaporation is 75 nm.

Within the wavelengths of 550–590 nm, there was evidence of a peak originating from the Si_3_N_4_ and Au interlayer, gradually widening and starting to overlap with the Au resonance peak as the thickness of the Au layer decreased. The optimal outcome, achieved with a 75 nm Au thickness, is presented in Figure 5, illustrating both the transmission and electric field enhancement results. The “hot spots” on the NHA are observed as red spots in the electric field enhancement results in Figure 5 (right). These simulation results provided the employed fabrication parameters.

**Figure 6:**
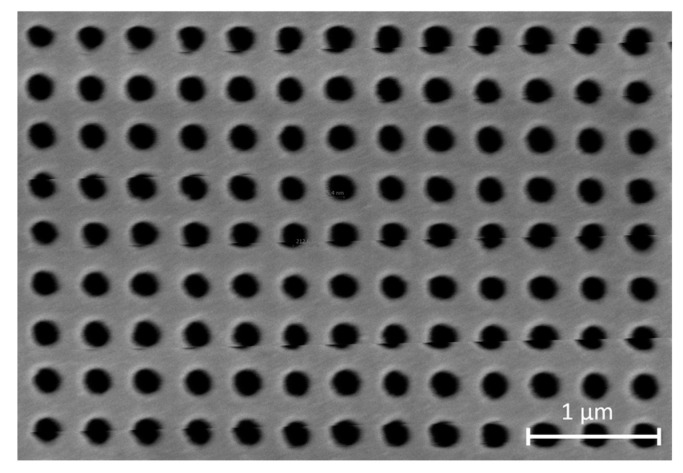
SEM image of an NHA sector, showing the periodicity of the structure.

NHA fabrication results were verified by scanning electron microscope (SEM) imaging. The diameter and period dimensions were measured to be 200 nm and 400 nm, respectively, as shown in Figure 6.

### 3.2. Control Response Demonstration

The performance of Au nanohole array-based LSPR sensors is significantly influenced by the refractive index, directly impacting sensitivity and detection capabilities. Changes in the refractive index of the surrounding medium lead to alterations in plasmonic properties, thereby affecting resonance wavelength and intensity, consequently influencing the sensor’s response to analytes. Higher variations in refractive index enhance sensitivity in detecting molecular binding events [2]. 

To validate the sensor’s performance and functionality for liquid-phase measurements with the new sensor cell, a comprehensive set of experiments was conducted. These experiments involved water–ethanol solutions with varied estimated refractive indices, determined based on the volume fractions of water and ethanol, as described by Arif et al. [20]. At a fixed wavelength of 600 nm, the estimated refractive indices for different water–ethanol volume fractions (1/0, 0.8/0.2, 0.6/0.4, 0.4/0.6, and 0.2/0.8) were 1.3322, 1.3378, 1.3436, 1.3495, and 1.3553, respectively. The volume of the solution applied was 200 µL, corresponding to ethanol concentrations of 0 mol/L, 5.4 mol/L, 14.5 mol/L, 32.6 mol/L, and 87.0 mol/L for water/ethanol volume fractions of 1/0, 0.8/0.2, 0.6/0.4, 0.4/0.6, and 0.2/0.8, respectively.

**Figure 7:**
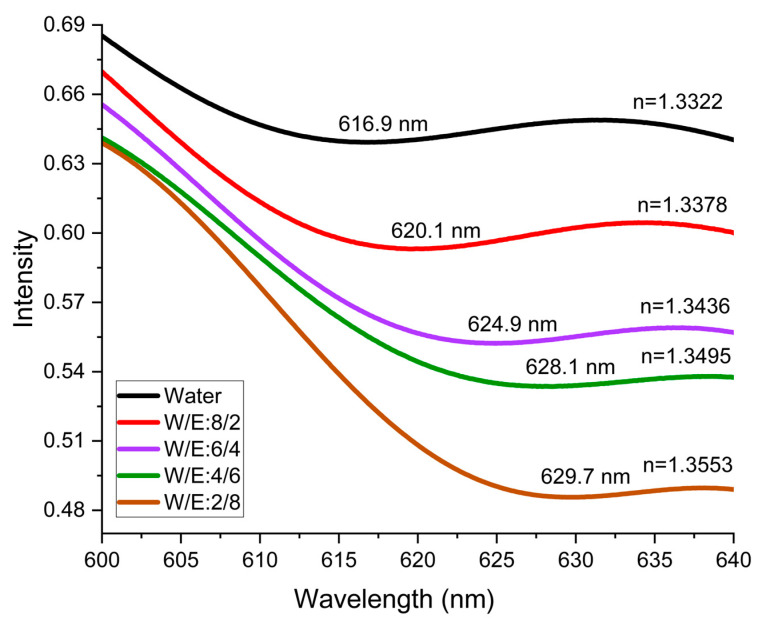
Spectrometer measurements of one trial from the NHA in different water/ethanol solutions of varied estimated refractive indices. The peak positions for each solution were identified as follows: 616.9 nm, 620.1 nm, 624.9 nm, 628.1 nm, and 629.7 nm corresponding to refractive indices of 1.3322, 1.3378, 1.3436, 1.3495, and 1.3553, respectively.

Figure 7 presents spectral fits for data acquired in one trial from the NHA covered by water–ethanol mixtures, serving as an illustrative example of the spectrometer measurements. In these fits, the peak positions for the different water–ethanol volume fractions (1/0, 0.8/0.2, 0.6/0.4, 0.4/0.6, and 0.2/0.8) were determined to be 616.9 nm, 620.1 nm, 624.9 nm, 628.1 nm, and 629.7 nm, respectively. Notably, a consistent shift in the peak position is observed with an increase in estimated refractive indices, confirming the sensor’s capability to detect and differentiate changes in the refractive indices of the test solutions.

**Figure 8:**
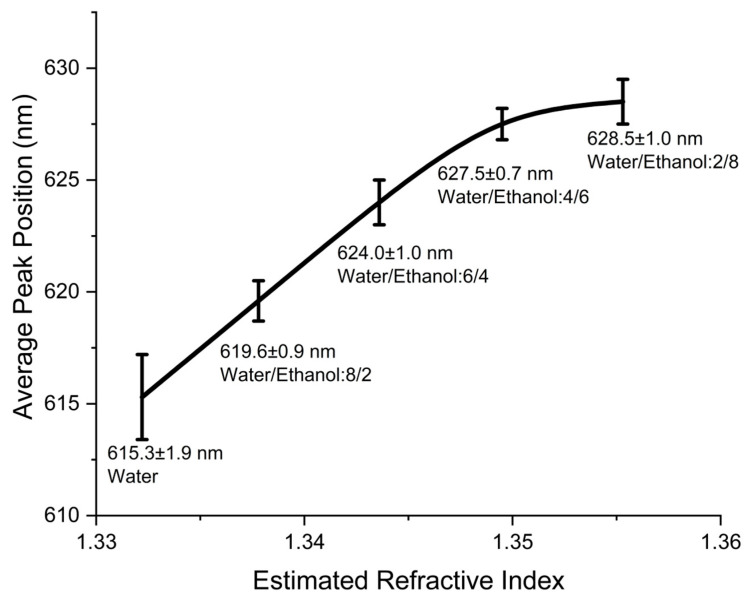
The relationship between the estimated refractive index and the average peak position in wavelength (nm) for the four NHA sectors. Across estimated refractive indices of 1.3322, 1.3378, 1.3436, 1.3495, and 1.3553, the corresponding average peak positions are 615.3 ± 1.9 nm, 619.6 ± 0.9 nm, 624.0 ± 1.0 nm, 627.5 ± 0.7 nm, and 628.5 ± 1.0 nm, respectively.

Measurements were recorded on all four NHA sectors, testing each sector three times to ensure reproducibility, resulting in 60 data points. Supplementary material Figures S1–S3 display additional spectrometer results for NHA sectors with water/ethanol data, providing insights into the concept and sensitivity. A consistent upward trend is observed in all NHA sectors as the refractive indices increase, affirming reproducibility. Figure 8 illustrates the collective average peak positions obtained by combining measurements from all NHA sectors under varying water–ethanol conditions. Across the estimated refractive indices of 1.3322, 1.3378, 1.3436, 1.3495, and 1.3553, the average peak positions for these NHA sectors were 615.3 ± 1.9 nm, 619.6 ± 0.9 nm, 624.0 ± 1.0 nm, 627.5 ± 0.7 nm, and 628.5 ± 1.0 nm, respectively. Notably, this pattern consistently aligns with the example data shown in Figure 7. As the ethanol volume ratio and estimated refractive indices increase, the peak position shifts toward red. These results confirm the sensor’s effectiveness and reproducibility within the new sensor cell, particularly in a liquid-phase environment. The successful demonstration validates its potential for robust and reliable performance, confirming its suitability for practical biomedical sensing applications.

## 4. Conclusions

The combination of plasmonic surfaces, their functionalization for monitoring biomolecular interactions, and subsequent plasmonic measurements hold significant promise in biomedical applications. This study demonstrates the simulation, design, and fabrication of NHA-based LSPR sensor platforms integrated into a PDMS channel for potential liquid-phase biomedical diagnostics. Future functionalization of the sensor surface using chemical methods, such as the self-assembled monolayer technique, can be devised. This platform presents evidence of the sensor’s effectiveness, providing reproducible data and enabling label-free analysis for diverse potential biomedical diagnostic applications. With adaptable surface modifications, this sensor platform can demonstrate adaptability, suggesting its potential utility across a spectrum of diseases in prospective biomedical diagnostics. FDTD simulations revealed that an optimal configuration for the platforms included a Au thickness of 75 nm, combined with a 5 nm Ti layer. Subsequently, NHA-based LSPR sensors were fabricated and successfully integrated with a PDMS well, allowing liquid sample introduction and conducting liquid-phase spectrometer measurements. The effectiveness of the PDMS microfluidic channel was further validated, confirming the sensor’s reliability through water–ethanol solution studies. This study reveals a multidisciplinary approach, encompassing plasmonic nanomaterials, biochemical elements, microfluidic technology, and optical components. The resulting sensor platform is positioned for target detection in biomedical diagnostics, offering versatility and robust performance across various applications.

## Data Availability

The data presented in this study are available in the article.

## Supplementary Materials

The following supporting information can be downloaded at: https://www.mdpi.com/article/10.3390/s24010186/s1, Figure S1: Spectrometer measurements of NHA2 in different water/ethanol solutions of varied estimated refractive indices. The peak positions for each solution were found at 616.8 nm, 619.5 nm, 623.6 nm, 626.1 nm, and 628.0 nm for refractive indices of 1.3322, 1.3378, 1.3436, 1.3495, and 1.3553, respectively; Figure S2: Spectrometer measurements of NHA3 in different water/ethanol solutions of varied estimated refractive indices. The peak positions for each solution were found at 617.2 nm, 620.0 nm, 624.9 nm, 627.7 nm, and 629.6 nm for refractive indices of 1.3322, 1.3378, 1.3436, 1.3495, and 1.3553, respectively; Figure S3: Spectrometer measurements of NHA4 in different water/ethanol solutions of varied estimated refractive indices. The peak positions for each solution were found at 616.7 nm, 620.2 nm, 624.6 nm, 627.0 nm, and 629.3 nm for refractive indices of 1.3322, 1.3378, 1.3436, 1.3495, and 1.3553, respectively; Table S1: An overview of plasmonic sensors: materials, performance, and applications.
