# Supplementary material for: Development of Liquid-Phase Plasmonic Sensor Platforms for Prospective Biomedical Applications
Source: Sensors (Basel). 2023 Dec 28;24(1):186. doi: 10.3390/s24010186 (PMC10781335; doi:10.3390/s24010186)
Supplement: Supplementary file 1 [file sensors-24-00186-s001.zip › sensors-2748696-supplementary.pdf]

sensors

Supplementary Materials

Development of Liquid-Phase Plasmonic Sensor Platforms for Prospective Biomedical Applications

Sezin Sayin [1,*], You Zhou [1], Sheng Wang [2], Andres Acosta Rodriguez [3] and Mona Zaghloul [1]

[1] Department of Electrical and Computer Engineering, School of Engineering and Applied Science, The George Washington University, Washington, DC 20052, USA
[2] Department of Biomedical Engineering, School of Engineering and Applied Science, The George Washington University, Washington, DC 20052, USA
[3] 3D Enviro, Barboursville, VA 22923, USA
* Correspondence: sezinsayin@email.gwu.edu

Table S1. An Overview of Plasmonic Sensors: Materials, Performance, and Applications.

Article	Sensor Material	Application	Sensor Performance
Pandey et al. [5]	Au Nanohole Array	Detection of acetone and ethanol vapors	500 nmol mol^{-1} to 320 µmol mol^{-1}, respectively.
Akib et al. [6]	BK7/Au/$PtSe_2$/Graphene) Coated SPR	Covid-19 detection	Increased sensitivity by (1 + 0.55) × graphene layers
Uddin et al. [7]	Si/$BaTiO_3$/Ag Based SPR	SARS-CoV-2 detection	130.4 degree/RIU
Qiu et al. [8]	Au Nanoislands	SARS-CoV-2 detection	Detection limit of 0.22 pM
Yang et al. [9]	Ag Nanotriangle Array	SARS-CoV-2 detection	Detection limit of 0.83 pM, 391 PFU/mL, and 625 PFU/mL
Yang and Li [10]	Hexagonal Au Nanohole Array	Investigation of sensitivity under different incident angles	1.5 times increased sensitivity in the sensor surface
Sun et al. [18]	Au Nanohole Array	Diagnosis of cystic fibrosis	Detection limit of 250 and 1000 nmol/mol

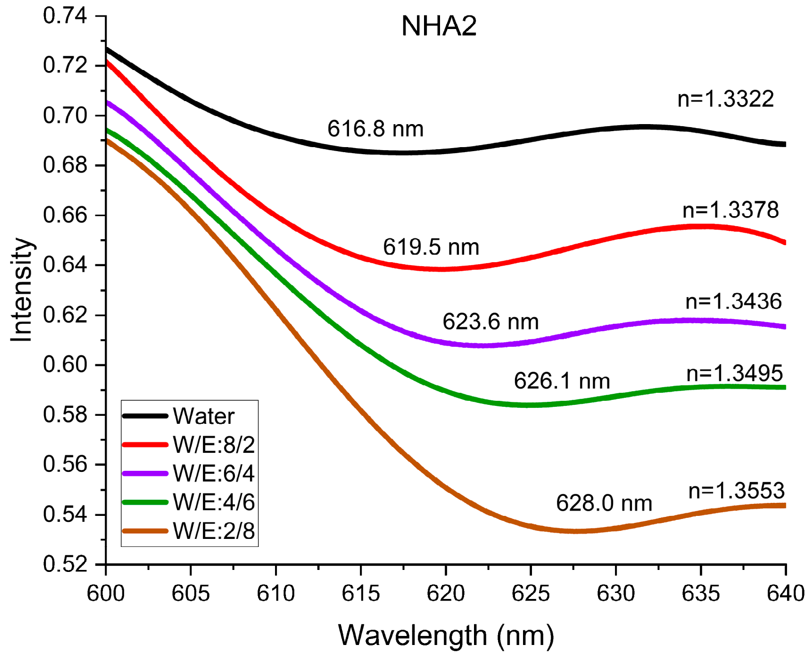

Figure S1. Spectrometer measurements of NHA2 in different water/ethanol solutions of varied estimated refractive indices. The peak positions for each solution were found 616.8 nm, 619.5 nm, 623.6 nm, 626.1 nm, and 628.0 nm for refractive indices of 1.3322, 1.3378, 1.3436, 1.3495, and 1.3553, respectively.

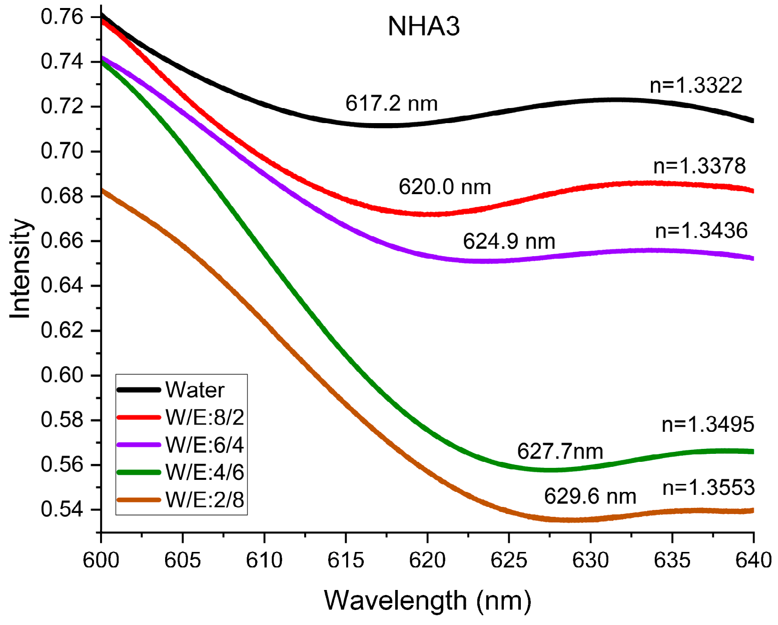

Figure S2. Spectrometer measurements of NHA3 in different water/ethanol solutions of varied estimated refractive indices. The peak positions for each solution were found 617.2 nm, 620.0 nm, 624.9 nm, 627.7 nm, and 629.6 nm for refractive indices of 1.3322, 1.3378, 1.3436, 1.3495, and 1.3553, respectively.

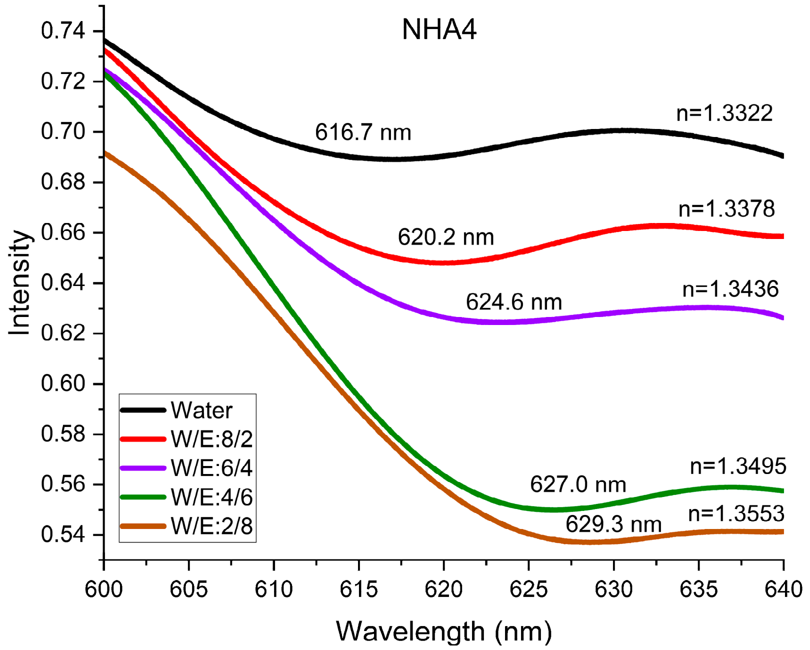

Figure S3. Spectrometer measurements of NHA4 in different water/ethanol solutions of varied estimated refractive indices. The peak positions for each solution were found 616.7 nm, 620.2 nm, 624.6 nm, 627.0 nm, and 629.3 nm for refractive indices of 1.3322, 1.3378, 1.3436, 1.3495, and 1.3553, respectively.
